# Extending beyond traditional forage: potential nutritional benefits of native plants in extreme arid insular regions

**DOI:** 10.3389/fpls.2024.1476809

**Published:** 2024-12-24

**Authors:** Raquel Pérez-Reverón, Adolfo Perdomo-González, Begoña de la Roza-Delgado, Covadonga Rodríguez, José A. Pérez-Pérez, Francisco J. Díaz-Peña

**Affiliations:** ^1^ Department of Animal Biology, Soil Science and Geology, University of La Laguna, San Cristóbal de La Laguna, Spain; ^2^ Department of Nutrition, Grasslands and Forages, Regional Institute for Research and Agro-Food Development, Villaviciosa, Spain

**Keywords:** alternative forage, crude protein, fatty acids, livestock productivity, mineral composition, nutritive value

## Abstract

The scarcity, unstable nutritional quality and environmental cost of imported forages in arid insular regions like Fuerteventura in the Canary Islands (Spain) need exploring sustainable local alternatives. This study evaluated the nutritional quality of twelve native and endemic plant species categorized into legumes, grasses, and a mixed group, cultivated under controlled conditions. The bromatological profiles, focusing on fiber, protein, lipids, and minerals, showed significant differences among plants in key parameters of forage quality: neutral detergent fiber (NDF; 24.2–71.3%), acid detergent fiber (ADF; 9.0–40.5%), acid detergent lignin (ADL; 2.0–15.8%), crude protein (CP; 6.1–20.9%), total lipids (TL; 1.5–6.3%), ash content (25.4–88.6%), enzymatic organic matter digestibility (EOMD; 5.9–10.9 MJ/kg), metabolizable energy (ME; 5.9–10.9 MJ/kg), and relative feed value (RFV; 74.8–317.9). Among lipids, all species had a high proportion of polyunsaturated fatty acids (PUFA; 34.7–63.1% of total fatty acids), mainly α-linolenic acid (ALA; 18:3 n-3; 24.8–54.4%) and linoleic acid (LA; 18:2 n-6; 6.4–25.0%). Other beneficial lipid molecules for animal health such as γ-linolenic acid (GLA; 18:3 n-6), stearidonic acid (SDA; 18:4n-3) and phytosterols (PTS) were detected in specific species. Mineral composition analysis revealed that only Ca, Na, Fe and Cu levels were near or above the established maximum tolerable levels (MTLs) in some species. According to literature, most of the species had a similar or slightly lower nutritional value compared to conventional forages such as alfalfa. Thus, the evaluated native species pool could serve as alternative feed for ruminants during forage shortages, suggesting their combined use to improve livestock health and product quality. This research emphasizes the untapped potential of native plant biodiversity to enhance sustainable agro-livestock practices in arid regions, supporting livestock nutrition and conserving unique botanical heritage.

## 1 Introduction

Forage crops play a crucial role in supporting livestock by significantly contributing to the health, productivity, and yield of animals (Chand et al., 2022). These crops have primarily comprised herbaceous legumes such as alfalfa (*Medicago sativa*) and various grass species including barley (*Hordeum vulgare*) and oats (*Avena sativa*). However, identifying alternative forages from native flora thriving in numerous arid and semi-arid regions where feed resources are scarce is receiving increasing attention in recent decades in response to the growing global food demand (e.g., Díaz et al., 2018; Louhaichi et al., 2021). These species usually exhibit greater adaptability than conventional crops to adverse conditions such as water stress and salinity, making them a valuable tool for adapting to projected climate scenarios of increased aridity while potentially meeting the nutritional needs of livestock over extended periods (Álvarez et al., 2007). Additionally, local forages can provide specific nutritional elements that positively impact the health and well-being of the herd, enhancing the value of derived meat and dairy products (El Shaer, 2010; Elgersma et al., 2013; Capstaff and Miller, 2018; Davis et al., 2022). Obtaining new forage crops requires an exhaustive study of the ecology and physiology of these plants, particularly their nutritional value, which can offer valuable insights into the most suitable candidates for domestication (Norman and Masters, 2023).

Considering that forages, whether fresh or preserved, are an essential part of the animal diet globally, providing high nutritional quality species is critical to ensure the health and productivity of livestock (Capstaff and Miller, 2018). Key parameters like crude protein (CP), digestibility, metabolizable energy (ME), fiber content, and lipid profile largely reflect the nutritional status of a forage (Clapham et al., 2005; Lee, 2018; Chand et al., 2022). These quality parameters vary significantly among different crops depending on their biochemical composition and prevailing agroclimatic conditions (Capstaff and Miller, 2018; Chand et al., 2022). Although there are notable differences in the nutritional value and palatability among forage plants from various botanical families, global data are sparse, and information is even more limited for native species (Lee, 2018; Louhaichi et al., 2021). In this context, a recent review indicates that legumes typically offer higher protein content than grasses, which may be more digestible, while information on other plant groups is scarce (Lee, 2018; Chand et al., 2022).

Forages are also a significant source of fatty acids (FAs) in ruminant diets, and its concentrations in different species are crucial for the quality of dairy and meat products (Elgersma et al., 2013; Davis et al., 2022). For example, a study evaluated the effects of dietary supplementation with fresh *Camelina sativa* in goats, finding that the milk contained higher levels of polyunsaturated fatty acids (PUFA) and conjugated linolenic acid (CLA) than traditional forages, and this milk also produced cheese with better sensory properties (Colonna et al., 2021). Therefore, increasing the available knowledge about forage compositional characteristics would aid in a more comprehensive understanding of its nutritional value for ruminants (Castro-Montoya and Dickhoefer, 2020; Norman and Masters, 2023).

In the Canary Islands, as in other arid and semi-arid regions of the world, there is a deficit in food production for livestock, becoming the most dependent Spanish region on imports for this sector (PFORCA, 2014; ISTAC, 2022). Particularly, in Fuerteventura Island, one of the more arid territories, nearly 30,000 tons of food are imported annually to feed around 84,000 goats and sheep (ISTAC, 2022), significantly impacting the environment due to the carbon footprint and greenhouse gas emissions from mainland transport. In contrast, this archipelago is an oceanic hotspot harboring an endemic plant diversity of more than 600 taxa, representing over 50% of the total native flora (Caujapé-Castells et al., 2022). Fuerteventura alone accounts for approximately 15% of the Canary Islands’ total endemic plant species (Biota, 2024). This taxonomic and genetic diversity provides numerous options with considerable potential as livestock forage. However, information about the performance and quality of these plants is limited and restricted to a few varieties (Méndez et al., 2003; Chinea et al., 2014; Álvarez et al., 2007; 2018). More specifically, to date, quantitative data on the lipid composition of native and endemic plant species are almost null (Guil-Guerrero et al., 2000a, b). The provision of new functional forage resources would allow increasing the pool of plant raw materials for rationing and opens the possibility of recovering abandoned farmland, contributing to environmental sustainability, soil conservation and the fight against desertification (Chinea et al., 2014; Hunter et al., 2017; Díaz et al., 2018). Given this potential, we pose the following questions: Will this biodiversity reservoir be able to meet the nutritional requirements of livestock? Will these species provide nutritional compounds that are potentially beneficial to animal and human health, thereby adding value to derived products such as milk and cheese?

Considering the above factors, this study aimed to assess the bromatological characteristics of several species from the native and endemic flora of Fuerteventura, focusing on key quality indicators like protein, fiber content, and lipid profiles, to identify forage crops with high nutritional quality traits that could enhance livestock productivity. The working hypothesis posits that evaluating nutritional qualities can serve as a tool to select the best single or combined candidate species from the native flora for potential cultivation in the arid and semi-arid zones of the Canary Archipelago.

## 2 Material and methods

### 2.1 Plant tissues sampling

**Figure 1:**
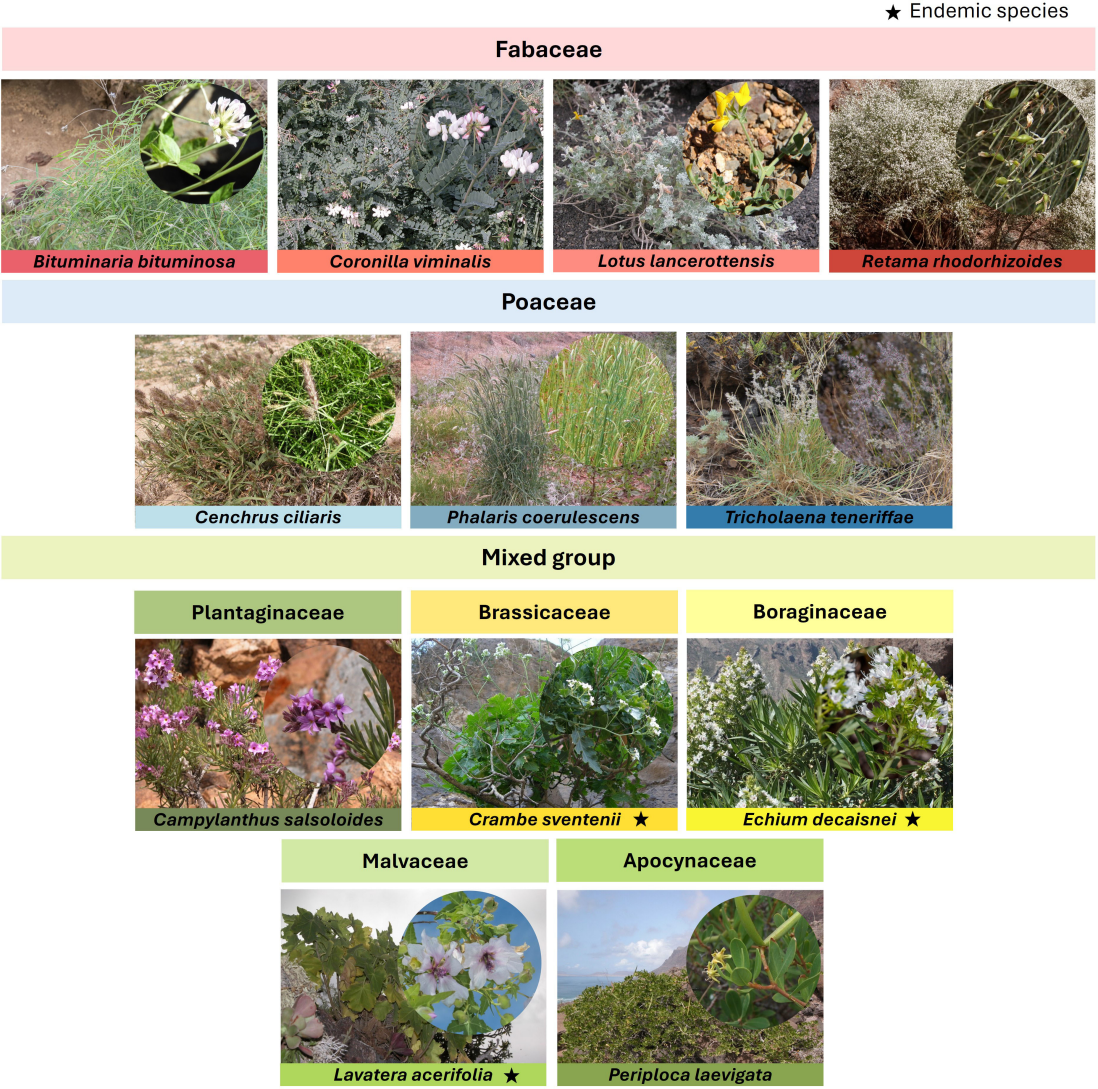
Studied plant species grouped by families. Photographs from Biota (2024) (*C. viminalis, L. lancerottensis, P. coerulescens, C. salsoloides, C. sventenii, E. decaisnei, L. acerifolia, P. laevigata,* by © G. García Casanova; *B. bituminosa, R. rhodorhizoides, C. ciliaris, P. laevigata* by © E. Ojeda-Land; *C. sventenii* by © S. Scholz) and Endémicas Canarias (2024) (*B. bituminosa, C. viminalis, R. rhodorhizoides, C. ciliaris, P. coerulescens, T. teneriffae* by © G. García Casanova).

The plants were grown at the facilities of a local government experimental farm located in the southwest of Fuerteventura Island (Canary Islands, Spain). This farm features a 1,200 m^2^ mesh nursery dedicated exclusively to the reproduction of native flora (**Supplementary Figure S1**). Twelve native and endemic species from the island of Fuerteventura, regarded in oral tradition as goat feed, were selected for the present study. These included four legumes (*Lotus lancerottensis, Bituminaria bituminosa, Coronilla viminalis*, and *Retama rhodorhizoides*), three grasses (*Cenchrus ciliaris, Phalaris coerulescens*, and *Tricholaena teneriffae*), and five other herbaceous and shrub species from different families denominated as mixed group (*Lavatera acerifolia, Periploca laevigata, Campylanthus salsoloides, Echium decaisnei*, and *Crambe sventenii*) (**Figure 1**).

All plants were germinated under controlled conditions and grown in a substrate made from a mix of Fuerteventura soil (Typic Torrifluvents; Soil Survey Staff, 2014) with a sandy-loam texture (clay 198.1 g/kg, silt 594.0 g/kg, and sand 207.9 g/kg), peat, and gravel in a 1:1:1 ratio, and irrigated with desalinated brackish water (EC_iw_ ~ 0.4 dS/m). The soil had a very low organic matter and nitrogen content, typical of arid region soils, requiring the use of basal fertilization (~ 6 g of NPK 17:6:14 fertilizer per 3 L volume container) to prevent nutritional deficiencies.

Plant tissue sampling took place in early November 2021 when the plants were between 10-18 months old. Samples (mainly leaves) from five individuals of each of the twelve selected species were collected. Once in the laboratory, the samples were pre-washed with distilled water, and divided into two subsamples. One of the subsamples was dried at 60°C for 72 hours and ground (1 mm) for fiber, protein, and minerals analysis, while the other subsample was stored at -80°C until their lipid analysis.

### 2.2 Analysis of plant tissues

The nutritive composition of the leaf tissues was determined using standard procedures (AOAC, 1990, 2000). The nutritional potential of the forages was assessed based on the levels of CP, ash, neutral detergent fiber (NDF), acid detergent fiber (ADF), acid detergent lignin (ADL), enzymatic digestibility of organic matter (EOMD), relative feed value (RFV), ME, lipid content, lipid classes and fatty acid profiles, and minerals. CP was determined by dried combustion and measurement in the resulting gases with a thermoconductivity cell from CN828 equipment (LECO Corporation, St. Joseph, MI, USA). The ash content after incineration at 550°C, NDF, and ADF were analyzed using the Van Soest fractionation analysis (Van Soest et al., 1991). ADL was extracted using 72% sulfuric acid after the ADF procedure described by Robertson and Van Soest (1981), and EOMD following de la Roza and Argamentería (1992). The RFV was calculated from the ADF and NDF as follows: RFV = [88.9 - (0.779 * ADF)] * [(120/NDF)/1.29] (Putnam et al., 2008). The ME was calculated from the ash content and the *in vitro* digestibility of organic matter (IVOMD) as follows: ME = K * (100 - ash) * (IVOMD/100), where K = 0.16 for forages and the IVOMD was predicted from EOMD values (ARC, 1984; de la Roza and Argamentería, 1992).

Lipid characterization was based on the content of total lipids (TL), lipid classes and FAs profiles. TL were extracted by homogenization in chloroform/methanol (2:1 v/v) containing 0.01% butylated hydroxytoluene (BHT) as an antioxidant according to the method described by Folch et al. (1957) with slight modifications (Christie and Han, 2010). The organic solvent was evaporated under a stream of nitrogen, and the lipid content was determined gravimetrically. The lipid residue was then resuspended in chloroform/methanol (2:1 v/v) containing 0.05% BHT and stored under an inert nitrogen atmosphere at -20°C until further analysis.

The lipid classes were determined by high-performance thin-layer chromatography (HPTLC) with dual one-dimensional development (Olsen and Henderson, 1989), using 1-propanol/chloroform/methyl acetate/methanol/potassium chloride at 0.25% (5:5:5:2:1.8, v/v) to separate the polar lipids (PL), and hexane/diethyl ether/acetic acid (20:5:0.5, v/v) for the neutral lipids (NL). Lipid classes were quantified by calibrated densitometry by means of a CAMAG TLC Visualizer dual-wavelength flying-spot scanner (Camag, Muttenz, Switzerland), as described by Reis et al. (2019). Lipid class identification was performed by comparison with external lipid standards (cod roe lipid extract and a mix of digalactosyldiacylglycerol (DGDG), monogalactosyldiacylglycerol (MGDG), and sulfoquinovosyldiacylglycerol (SQDG) (Avanti Polar Lipids, Inc., Alabaster, Alabama, USA) placed on the same HPTLC plate (**Supplementary Figure S2**). The proportion of each lipid class was expressed as a percentage of the TL in the analyzed sample.

Another aliquot of TL was subjected to acid-catalyzed transesterification according to Christie and Han (2010). The resulting fatty acid methyl esters (FAMEs) were purified by thin-layer chromatography (TLC) and separated and quantified using a TRACE-GC Ultra gas chromatograph (Thermo Fisher Scientific Inc., Milan, Italy) under the chromatographic conditions described in Galindo et al. (2022). Individual FAMEs were identified by reference to a well-characterized commercial standard mixture (Mix C4-C24 and PUFA No. 3 from menhaden oil (Supelco Inc., Merck KGaA, Darmstadt, Germany)), and their relative abundance was expressed as a percentage of the total FAs in the analyzed sample (**Supplementary Figure S3**). Before transesterification, nonadecanoic acid (19:0) was added to the lipid fraction as an internal standard. When necessary, the identity of FAME was confirmed by GC-MS (DSQII, Thermo Scientific), under the same chromatographic conditions.

Nutritional quality of plant FA composition was assessed by calculating the index of atherogenicity (IA), the index of thrombogenicity (IT) following Ulbricht and Southgate (1991), and the hypocholesterolemic (h) to hypercholesterolemic (H) FAs ratio (hH) as described by Santos-Silva et al. (2002):


IA=[12:0+(4×14:0)+16:0]/(∑MUFA+∑n−6 PUFA+∑n−3 PUFA)

IT=(14:0+16:0+18:0)/(0.5×∑MUFA+0.5×∑n−6 PUFA+3×∑n−3 PUFA+n−3/n−6 ratio)

hH=(18:1n−9+18:2n−6+20:4n−6+18:3n−3+20:5n−3+22:5n−3+22:6n−3)/(14:0+16:0)

The elements analyzed for the study of mineral composition were N, P, K, Ca, Mg, Na, Fe, Mn, Cu, Zn, and B. All elements were determined by digestion combined with inductively coupled plasma mass spectrometry (ICP-MS), except N, which was measured using LECO CN828 equipment. The concentrations of these elements were compared with the established maximum tolerable levels (MTLs), defined as the highest level of a particular element in an animal’s diet over a specified period that does not lead to health deterioration or a decrease in animal performance (NRC, 2005).

### 2.3 Statistical analysis

Statistical methods were implemented using R version 4.3.0 (R Core Team, 2023) and IBM SPSS Statistics V26.0. The significance level for all tests was set at *p*< 0.05. Prior to each analysis, the assumptions of normality (Shapiro-Wilk test) and homogeneity of variance (Levene’s test) were verified within groups, and, where necessary, appropriate variance stabilizing transformations were performed. For all parameters, differences between the plant species were evaluated using a one-way ANOVA followed by the Tukey HSD *post-hoc* test. When transformations failed, Welch and Brown-Forsythe test followed by the Dunnett T3 test, or non-parametric Kruskal Wallis and Mann-Whitney U tests with Bonferroni adjustment were performed. Principal component analysis (PCA) for the main nutritional parameters of plant species was carried out. Two hierarchical cluster heatmaps were created using Euclidean distance, one for the lipid classes and the other for the main FAs groups, to identify patterns among species.

## 3 Results

### 3.1 Forage fiber, protein and ash contents

**Table 1** presents the main nutritional parameters related to fiber, protein and ash contents of the analyzed plant species. Levels of NDF, ADF, and ADL varied strongly between families and species (**Table 1**). Poaceae species exhibited higher average values of NDF and ADF compared to the other groups (e.g., 64.8, 41.4 and 28.0% for NDF in Poaceae, Fabaceae and mixed groups, respectively). *T. teneriffae* was the grass species with the highest concentrations of NDF and ADF among all analyzed plants. In contrast, ADL levels were up to two-three times higher in *R. rhodorhizoides* and *E. decaisnei* than in the other species. Content of CP varied significantly from ~ 6% (*T. teneriffae*) to ~ 32% (*P. coerulescens)*, both from the Poaceae family. Generally, legumes surpassed grasses in terms of CP, except for *P. coerulescens* which presented the highest proportions of all analyzed species. Species within the mixed group showed CP levels close to those of legumes (average values 14.2 vs. 17.5%), with *C. salsoloides* reaching an average concentration of 18.9% (**Table 1**). Ash content ranged from ~ 5% (*R. rhodorhizoides*) to 19% (*C. sventenii*). The average EOMD for all species in the mixed group was 80.0%, which was significantly higher compared to legumes (64.1%) and grasses (40.1%). *C. sventenii*, *B. bituminosa*, and *C. ciliaris* notably stood out within their respective groups. The energy potential of forage, calculated as ME, ranged from 8.1 to 8.6 MJ/kg DM on average per group. Among all studied species, *B. bituminosa* recorded the highest significant values (~ 10.9 MJ/kg DM), unlike specimens of *R. rhodorhizoides* which averaged 5.9 MJ/kg DM. Levels of RFV ranged from 75 in *T. teneriffae* to 318 in *C. salsoloides*. The mixed group had the highest average RFV indices (260), followed by Fabaceae (165) and Poaceae (108).

**Table 1:** Main nutritional parameters related to fiber, protein and ash contents of the plant species grouped into three categories by family: Fabaceae, Poaceae, and a mixed group (Plantaginaceae, Brassicaceae, Boraginaceae, Malvaceae and Apocynaceae).

Family	Species	NDF	ADF	ADL	CP	Ash	EOMD	ME	RFV
		% DM basis						MJ/kg DM	
Fabaceae	*B. bituminosa*	38.4 ± 0.8 b	19.0 ± 0.8 a	4.3 ± 0.4 a	20.9 ± 0.6 d	9.6 ± 0.1 b	76.8 ± 3.6 c	10.9 ± 0.9 c	179.7 ± 5.4 a
	*C. viminalis*	37.0 ± 0.5 b	19.9 ± 0.4 a	4.8 ± 0.1 a	19.7 ± 0.5 c	9.3 ± 3.6 ab	66.9 ± 4.4 b	8.8 ± 1.0 b	184.7 ± 2.0 a
	*L. lancerottensis*	35.0 ± 3.4 a	21.3 ± 1.3 ab	6.2 ± 1.1 b	15.0 ± 0.2 b	12.8 ± 0.4 b	69.8 ± 1.3 b	8.7 ± 0.5 b	193.5 ± 17.6 ab
	*R. rhodorhizoides*	55.5 ± 4.9 c	35.3 ± 1.0 c	14.3 ± 0.7 c	14.1 ± 0.2 a	5.4 ± 1.5 a	42.8 ± 2.0 a	5.9 ± 0.8 a	193.5 ± 17.6 b
Poaceae	*C. ciliaris*	64.5 ± 4.4 b	38.2 ± 1.3 b	5.5 ± 0.8 ab	7.6 ± 0.1 b	11.5 ± 1.6 bc	55.9 ± 10.0 c	9.4 ± 0.6 b	85.6 ± 5.7 b
	*P. coerulescens*	58.5 ± 0.5 a	28.9 ± 0.6 a	4.5 ± 0.1 a	31.5 ± 0.2 c	13.3 ± 0.4 c	38.7 ± 0.5 b	7.8 ± 1.1 a	105.6 ± 1.2 c
	*T. teneriffae*	71.3 ± 1.1 c	40.5 ± 0.4 c	6.3 ± 0.4 b	6.1 ± 0.3 a	7.5 ± 0.2 a	25.4 ± 6.2 a	7.6 ± 0.4 a	74.8 ± 1.4 a
Mixed group	*C. salsoloides*	22.7 ± 1.6 a	14.8 ± 1.9 b	6.8 ± 1.4 b	18.9 ± 0.2 e	8.5 ± 1.0 a	85.8 ± 1.0 c	8.6 ± 0.2 b	317.9 ± 21.8 c
	*C. sventenii*	24.7 ± 1.6 b	14.9 ± 0.6 b	2.9 ± 0.8 a	14.2 ± 0.2 d	19.1 ± 1.3 c	88.6 ± 3.6 d	7.6 ± 0.2 a	292.4 ± 18.3 b
	*E. decaisnei*	24.2 ± 3.4 b	24.8 ± 0.3 c	15.8 ± 0.6 d	13.5 ± 0.2 c	9.6 ± 0.7 ab	80.9 ± 1.2 b	7.4 ± 0.2 a	272.0 ± 40.4 b
	*L. acerifolia*	27.0 ± 3.2 b	9.0 ± 0.5 a	2.0 ± 0.3 a	11.5 ± 0.1 a	12.4 ± 0.6 b	78.8 ± 4.7 b	7.8 ± 0.4 a	285.5 ± 33.7 b
	*P. laevigata*	41.5 ± 3.5 c	25.0 ± 1.5 c	8.3 ± 0.2 c	12.7 ± 0.2 b	8.2 ± 0.6 a	65.8 ± 3.6 a	8.7 ± 0.3 b	156.8 ± 14.4 a

Data are mean ± standard deviation (n=5); different letters indicate significant differences among species within each group (*p*< 0.05).

NDF, neutral detergent fiber; ADF, acid detergent fiber; ADL, acid detergent lignin; CP, crude protein; EOMD, enzymatic organic matter digestibility; ME, metabolizable energy; RFV, relative feed value; DM, dry matter.

### 3.2 Forage lipid composition

#### 3.2.1 Total lipid content

**Figure 2:**
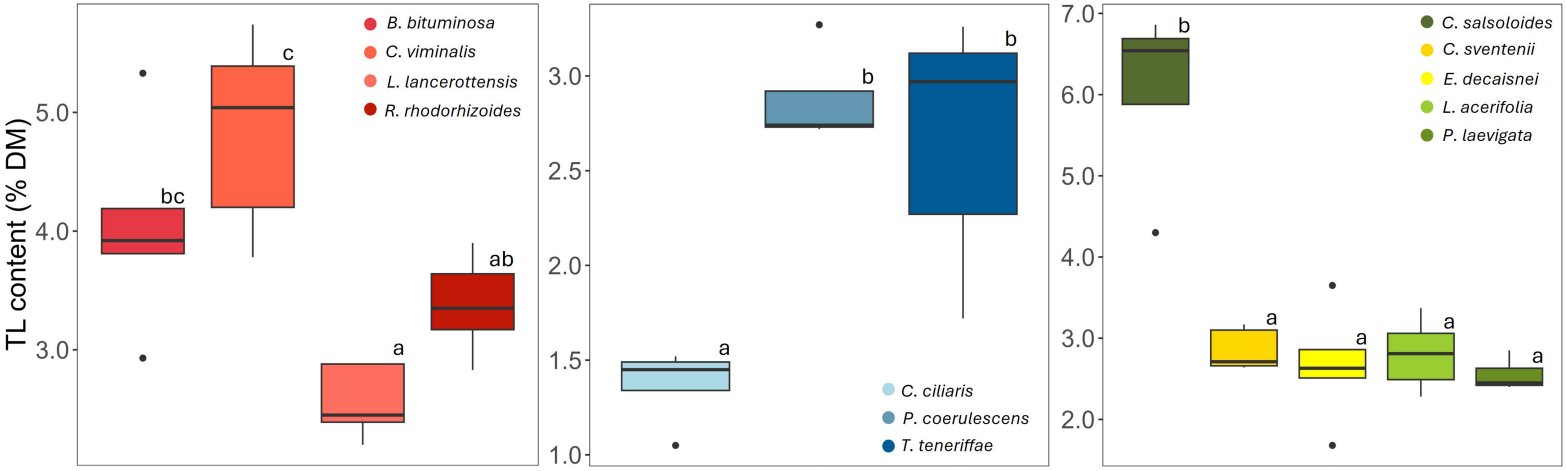
Total lipid (TL) content (% of DM) of the plant species grouped into three categories by family: Fabaceae, Poaceae, and a mixed group (Plantaginaceae, Brassicaceae, Boraginaceae, Malvaceae and Apocynaceae); n=5 for each boxplot; boxes represent the middle 50% of the data (interquartile range), with the line inside the box representing the median; the whiskers indicate the range of the data excluding outliers represented as isolated points; different letters indicate significant differences among species within each group (*p*< 0.05).

The total lipid (TL) content of the analyzed species ranged between 1.5 and 6.3% of DM (**Figure 2**). Within the legume group, TL tended to be higher in *C. viminalis* compared to the rest (> 5% vs. 3-4%, respectively) whereas all grasses presented values from 1.5 to 3% of DM, with significantly lower levels in *C. ciliaris*. *C. salsoloides* showed the highest TL proportions (> 6%) of all analyzed plants.

**Figure 3:**
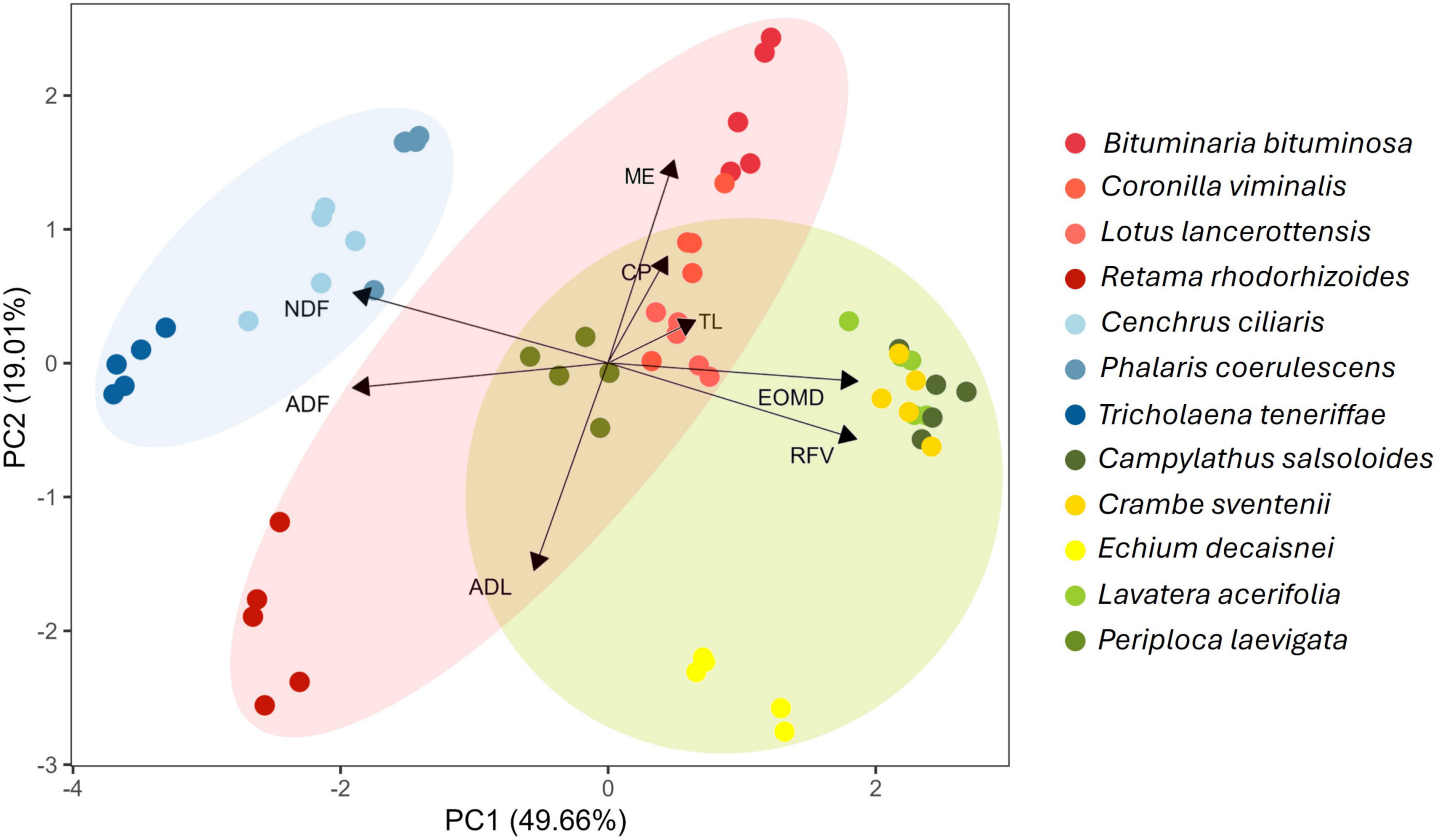
PCA of the main nutritional parameters in plant species analyzed. ADL, acid detergent lignin; ADF, acid detergent fiber; CP, crude protein; DM, dry matter; EOMD, enzymatic organic matter digestibility; ME, metabolizable energy; NDF, neutral detergent fiber; RFV, relative feed value; TL, total lipid content.

The multivariate PCA depicted in **Figure 3** combines the above-mentioned nutritional factors and confirms the observations regarding nutritional differences between the analyzed species. Based on this analysis, 68.67% of the variance was explained by the two principal components. The first component (PC1) showed a strong positive influence with RFV and EOMD (score = 1.936 and 1.920), while ADF and NDF were the variables with the highest negative values (-1.956 and -1.954, respectively). In the second component (PC2), the variables with the highest weight were ME (score = 1.758) and ADL (score = -1.776). Poaceae species were clearly differentiated from the others, mainly due to the high levels of NDF and ADF. Species from the mixed group showed a closer relationship with EOMD and RFV, except for *P. laevigata* and *E. decaisnei*. This distinction was mainly due to higher levels of ADL and ADF in those two species. Most legumes showed a positive correlation with ME and CP, with the exception of *R. rhodorhizoides*, which also exhibited high levels of ADL and ADF (**Figure 3**).

#### 3.2.2 Lipid classes profiles

The total lipids of the analyzed species presented highly variable proportions of total polar lipids (TPL; from 38 to 59% of TL) and total neutral lipids (TNL; from 41 to 62% of TL) (**Supplementary Table S1**). Concentration of TPL was greater than TNL, except *B. bituminosa*, in *P. coerulescens* (Poaceae) and *C. salsoloides* (Boraginaceae) (**Supplementary Table S1**). Among the PL, three main fractions clearly stood out: MGDG (8.4-22.5% of TL), DGDG (8.8-17.9% of TL), and SQDG (5.3-12.3% TL). In legumes MGDG levels were approximately 7.5-fold higher than in grasses, whereas SQDG + PE were up to four times lower in legumes. By contrast, DGDG proportions were similar in both families. Regarding TNL, free fatty acids (FFA) were generally more abundant in legumes than in grasses (**Supplementary Table S1**). Significant quantities of phytosterols (PTS) were present in all analyzed species (> 5% of TL), particularly in *R. rhodorhizoides, C. ciliaris*, *T. teneriffae*, and *C. seventenii* where average amounts up 10% of TL were registered.

**Figure 4:**
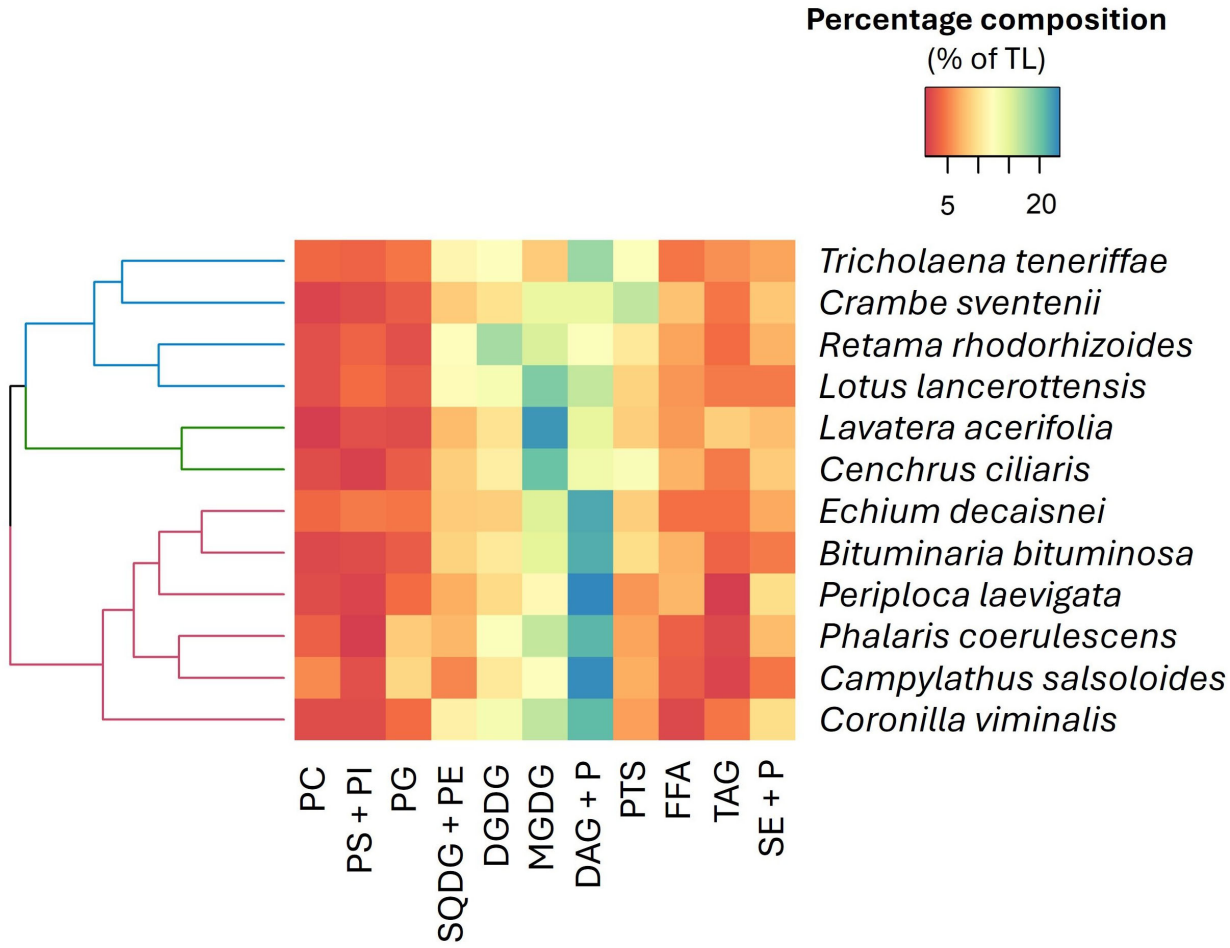
Hierarchically clustered heatmap (using Euclidean distance) according to the lipid class composition. The color gradient from blue to red represents the mean proportions of each lipid fraction (n=5), with blue indicating higher proportions and red denoting lower proportions within the dataset. PC, phosphatidylcholine; PS, phosphatidylserine; PI, phosphatidylinositol; PG, phosphatidylglycerol; SQDG, sulfoquinovosyldiacylglycerol; PE, phosphatidylethanolamine; DGDG, digalactosyldiacylglycerol; MGDG, monogalactosyldiacylglycerol; DAG, diacylglycerols; P, pigments; PTS, phytosterols; FFA, free fatty acids; TAG, triacylglycerols; SE, sterol esters.

A heatmap of lipid classes (**Figure 4**) identified three clusters based on the abundance patterns in the analyzed plants. Species in cluster 1 (e.g., *C. sventenii* and *R. rhodorhizoides*) were dominated by SQDG + phosphatidylethanolamine (PE), DGDG, and PTS compounds. Cluster 2 (i.e., *L. acerifolia* and *C. ciliaris*) stood out for their high contents of MGDG. The abundance of phosphatidylcholine (PC), phosphatidylglycerol (PG), and diacylglycerols (DAG) + pigments (P) was characteristic of species in cluster 3 (e.g., *P. coerulescens* and *C. salsoloides*).

#### 3.2.3 Fatty acids profiles

Fatty acids profiles and quality indices for the studied plant species are shown in **Supplementary Tables S2**, **S3**, respectively. In all species, FA groups were predominantly represented by PUFA (from 46.5 to 63.1% of total FA), followed by saturated fatty acids (SFA; from 24.3 to 33.7% of total FA), and to a lesser extent, monounsaturated fatty acids (MUFA; from 7.1 to 18.4% of total FA). An exception was *E. decaisnei*, which had higher levels of SFA than PUFA (43.6 vs. 34.7%, respectively). Palmitic acid (16:0) was the most abundant saturated fatty acid, representing more than 50% of the SFA in all cases. Within PUFA, linoleic acid (LA; 18:2 n-6) and alpha-linolenic acid (ALA; 18:3 n-3) dominated, comprising up to 25% and 55% of total FAs, respectively (**Supplementary Table S2**). It is noteworthy that *E. decaisnei* was the only species where gamma-linolenic acid (GLA; 18:3 n-6) was detected, with an average value of 0.8 ± 0.2% of total FA and where the amount of stearidonic acid (SDA; 18:4n-3) was also prominent (2.3± 0.3% of total FA), compared to the other species (**Supplementary Table S2**). N-3/n-6 PUFA ratio was > 1 in all the analyzed species, being especially relevant in *B. bituminosa*, *P. coerulescens*, and *C. salsoloides* (5.4 ± 0.3, 8.1 ± 0.5 and 5.1 ± 0.7, respectively) (**Supplementary Table S2**).

**Figure 5:**
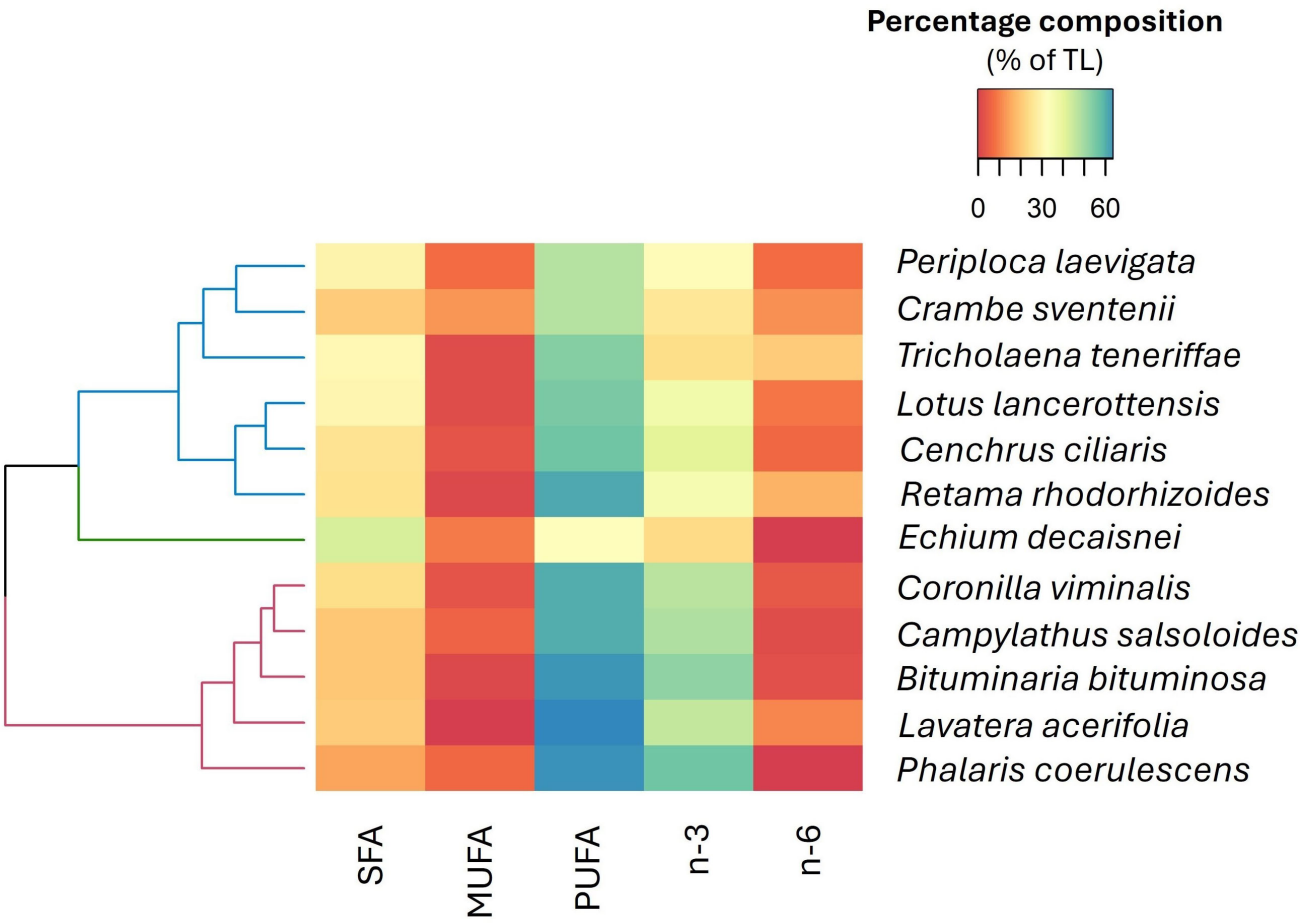
Hierarchically clustered heatmap (using Euclidean distance) according to the fatty acid composition. This analysis includes saturated (SFA), monounsaturated (MUFA), polyunsaturated (PUFA), omega-3 (n-3) and omega-6 (n-6). The color gradient from blue to red represents the mean proportions of each fatty acid group (n=5), with blue indicating higher proportions and red denoting lower proportions within the dataset.

The heatmap of FA families highlighted three familial clusters (**Figure 5**). In cluster 1, high concentrations of SFA and MUFA dominated, with species such as *T. teneriffae* and *C. sventenii* exhibiting the highest values, respectively. Cluster 2 is distinguished by an abundance of SFA, with *E. decaisnei* being the species with the greatest representation of this component. Finally, cluster 3 is characterized by a predominance of PUFA, n-3, and n-6 (e.g, *L. acerifolia* and *P. coesulescens)* displaying the highest average concentrations.

*B. bituminosa*, *R. rhodorhizoides*, *P. coerulescens*, and *C. salsoloides* showed the lowest IA and IT indices (~ 0.3 and 0.1, respectively) of all species. Regarding the hH ratio, *R. rhodorhizoides*, *T. teneriffae*, *C. sventenii*, and *P. laevigata* presented ratios ≥ 1, with the legume species showing the highest hH value at 1.7 (**Supplementary Table S3**).

### 3.3 Forage mineral composition

The mean values of macronutrients showed significant variations among species (**Table 2**). Thus, Ca concentrations were higher in legumes than in grasses (8.8 and 4.7 g/kg, respectively), but the highest concentrations were reached in *E. decaisnei* and *P. laevigata* (up to 22.7 and 34.2 g/kg, respectively). Regardless of the plant group, Mg levels remained low with average values around 3 g/kg. Potassium dominated in *E. decaisnei* and *C. viminalis*, not exceeding 35 g/kg in any specimen. The lowest K values were found in *C. salsoloides*, *C. sventenii*, *R. rhodorhizoides*, and *T. teneriffae* (ranging from 3.6 to 8 g/kg). Sodium concentrations were high for *E. decaisnei* and *P. laevigata* (~ 26 g/kg), followed by two legumes species *C. viminalis* and *L. lancerottensis*. not exceeding 5 g/kg in the remaining species. Content of P was similar in all species, ranging between 0.3 and 2.3 g/kg, except in *E. decaisnei* which presented the highest average value of 3.4 g/kg. Nitrogen followed the same pattern as CP, peaking in *P. coerulescens* (**Table 2**).

**Table 2:** Tissue macronutrient concentration of the plant species grouped into three categories by family: Fabaceae, Poaceae, and a mixed group (Plantaginaceae, Brassicaceae, Boraginaceae, Malvaceae and Apocynaceae).

Family	Species	Macronutrients (g/kg)					
		Ca	Mg	K	Na	P	N
Fabaceae	*B. bituminosa*	7.2 ± 0.1 b	2.8 ± 0.0 b	18.7 ± 0.2 c	1.8 ± 0.0 a	2.3 ± 0.0 b	33.4 ± 0.9 d
	*C. viminalis*	13.7 ± 0.4 d	3.8 ± 0.1 c	27.9 ± 0.7 d	16.4 ± 0.6 c	2.1 ± 0.1 b	31.5 ± 0.7 cd
	*L. lancerottensis*	12.7 ± 0.4 c	3.3 ± 0.1 c	13.3 ± 0.4 b	10.1 ± 0.4 b	1.8 ± 0.0 b	24.0 ± 0.4 b
	*R. rhodorhizoides*	1.7 ± 0.1 a	0.8 ± 0.1 a	4.7 ± 0.4 a	1.4 ± 0.2 a	0.5 ± 0.0 a	22.5 ± 0.4 a
Poaceae	*C. ciliaris*	3.4 ± 1.0 b	3.1 ± 0.2 b	14.4 ± 0.6 b	3.6 ± 0.1 b	1.2 ± 0.2 a	12.2 ± 0.2 b
	*P. coerulescens*	1.0 ± 0.1 a	0.9 ± 0.1 a	19.1 ± 1.9 c	2.0 ± 0.2 a	1.1 ± 0.2 a	50.4 ± 0.3 c
	*T. teneriffae*	5.7 ± 0.4 c	3.2 ± 0.4 b	8.0 ± 0.5 a	2.2 ± 0.1 a	1.7 ± 0.1 b	9.7 ± 0.4 a
Mixed group	*C. salsoloides*	0.7 ± 0.2 a	0.3 ± 0.0 a	3.6 ± 0.4 a	3.2 ± 0.6 a	0.3 ± 0.0 a	30.2 ± 0.2 e
	*C. sventenii*	4.1 ± 0.6 b	0.8 ± 0.1 a	5.2 ± 1.1 b	2.9 ± 0.4 a	0.7 ± 0.0 a	22.8 ± 0.4 d
	*E. decaisnei*	22.7 ± 4.0 d	3.2 ± 0.1 b	34.9 ± 1.3 d	27.5 ± 3.2 c	3.4 ± 0.2 c	21.6 ± 0.3 bc
	*L. acerifolia*	34.2 ± 2.2 e	3.4 ± 0.2 b	14.1 ± 0.8 c	5.1 ± 0.4 b	1.1 ± 0.1 b	18.5 ± 0.1 a
	*P. laevigata*	11.7 ± 0.2 c	3.4 ± 0.1 b	17.0 ± 0.7 c	24.4 ± 1.0 c	1.5 ± 0.0 b	20.3 ± 0.3 b

Data are mean ± standard deviation (n=5); different letters indicate significant differences among species within each group (*p*< 0.05).

The mean micronutrient concentrations in the collected plant species are presented in **Table 3**. Similarly to macroelements, significant differences were detected between groups and species. Iron levels showed high variability, with differences of up to approximately two orders of magnitude, led by *L. lancerottensis* and at the opposite end *C. salsoloides* (803.6 vs. 9.3 mg/kg). In most species, average Mn values fluctuated between ~ 46 and 81 mg/kg, except for *R. rhodorhizoides*, *P. coerulescens*, *C. salsoloides* and *C. sventenii* where it accounted for 1-2 mg/kg. Copper and Zn followed similar trends, with the highest concentrations in legumes and mixed group (average ~ 37.0 and 45.3 mg/kg for Cu and Zn, respectively). In both cases, *E. decaisnei* presented the highest concentrations of these elements. Levels of B also exhibited high variability, ranging from less than 10 mg/kg in grasses such as *P. coerulescens* and *T. teneriffae* to levels exceeding 100 mg/kg in the legume *C. viminalis* (**Table 3**).

**Table 3:** Tissue micronutrient concentrations of the plant species grouped into three categories by family: Fabaceae, Poaceae, and a mixed group (Plantaginaceae, Brassicaceae, Boraginaceae, Malvaceae and Apocynaceae).

Family	Species	Micronutrients (mg/kg)				
		Fe	Mn	Cu	Zn	B
Fabaceae	*B. bituminosa*	216.0 ± 2.6 c	56.7 ± 0.4 d	35.2 ± 0.9 b	38.9 ± 1.3 b	77.2 ± 1.3 c
	*C. viminalis*	84.9 ± 7.1 b	41.4 ± 4.4 b	33.8 ± 0.7 b	37.1 ± 3.1 b	109.4 ± 3.8 d
	*L. lancerottensis*	803.6 ± 20.1 d	50.1 ± 0.8 c	33.7 ± 0.9 b	47.4 ± 2.6 c	68.4 ± 1.8 b
	*R. rhodorhizoides*	15.9 ± 3.5 a	1.1 ± 0.1 a	4.9 ± 0.8 a	0.1 ± 0.0 a	17.9 ± 2.7 a
Poaceae	*C. ciliaris*	327.1 ± 12.7 b	81.4 ± 3.1 c	30.9 ± 2.6 c	31.2 ± 4.4 c	26.6 ± 4.3 b
	*P. coerulescens*	105.6 ± 11.7 a	1.7 ± 0.2 a	1.2 ± 0.3 a	2.2 ± 0.5 a	7.6 ± 1.0 a
	*T. teneriffae*	359.0 ± 26.0 c	31.8 ± 2.2 b	13.2 ± 1.4 b	11.3 ± 2.2 b	7.0 ± 2.1 a
Mixed group	*C. salsoloides*	9.3 ± 0.9 a	1.1 ± 0.1 a	5.5 ± 0.6 a	0.2 ± 0.0 a	3.9 ± 0.2 a
	*C. sventenii*	67.3 ± 4.4 b	1.7 ± 0.2 a	4.3 ± 1.8 a	0.1 ± 0.0 a	12.7 ± 2.7 b
	*E. decaisnei*	363.9 ± 16.0 d	65.1 ± 3.4 d	51.4 ± 6.2 d	56.4 ± 3.8 c	2.7 ± 0.7 a
	*L. acerifolia*	280.3 ± 7.5 c	45.6 ± 4.2 b	30.1 ± 3.1 b	45.5 ± 2.6 b	87.3 ± 7.9 c
	*P. laevigata*	446.1 ± 20.0 e	53.6 ± 1.3 c	37.9 ± 3.3 c	46.4 ± 3.9 b	98.8 ± 4.1 cd

Data are mean ± standard deviation (n=5); different letters indicate significant differences among species within each group (*p*< 0.05).

## 4 Discussion

### 4.1 Implications of the fiber, protein and ash profile on forage quality

Forage quality generally improves with increases in CP, EOMD, RFV, and ME, and reductions in NDF, ADF, and ash content (Suyama et al., 2007a; Díaz et al., 2018; Pirasteh-Anosheh et al., 2023). The levels of NDF, ADF and ash found in our present work coincide largely with those reported by other authors for species such as *B. bituminosa*, *C. viminales*, *L. lancerottensis* and *E. decaisnei* under natural conditions (Chinea et al., 2014). However, CP values were slightly higher than those previously reported, possibly due to the more favorable nursery conditions. When compared to a protein-rich forage like alfalfa (15-20% CP; Annicchiarico et al., 2015; Yang et al., 2021), four of the studied species (*L. lancerottensis*, *B. bituminosa*, *C. viminalis* and *C. salsoloides*) exhibited CP levels within this range (**Table 1**), demonstrating their potential as high-quality forage. The critical threshold of CP required for maintaining an adult non-pregnant sheep is approximately 7-9% (Pirasteh-Anosheh et al., 2023), and all examined native species, except *T. teneriffae*, surpassed this minimum, reinforcing their suitability for forage based on nitrogen and protein benchmarks.

The fiber percentages in our study (24.2-64.5% NDF and 9.0-40.5% ADF) also reflect the nutritional diversity within the studied forages. Eight out of twelve native species analyzed had an NDF content below 45%, and nine out of twelve had an ADF content below 35%, positioning them well within the ranges considered suitable for high-quality forage (Pirasteh-Anosheh et al., 2023). This has a positive impact on intake, energy density, milk production, cattle health, and feed cost (Christensen et al., 2015; Anderson et al., 2023). All forages had acceptable levels of ME (i.e., > 7 MJ/kg DM in most cases) for beef cattle and goats fed at a maintenance energy level (NRC, 1996). Considering that a 53 kg sheep needs forage with an ME content of 10-12 MJ/kg (Pirasteh-Anosheh et al., 2023)., only *B. bituminosa* met this requirement. This matches findings from Pirasteh-Anosheh et al. (2023) who reported that none of the selected halophyte species reached the required ME content.

The most valuable species in terms of forage quality within legumes were *B. bituminosa* and *C. viminalis*, both *P. coerulescens* and *C. ciliaris* within grasses, and *C. salsoloides*, *E. decaisnei* and *C. sventenii* for the mix group. Another criterion based on the quality parameters analyzed indicates that legumes can be classified as “premium” quality, grasses as “good-utility” and the rest between “premium” and “good” (Putnam et al., 2008; USDA Agricultural Marketing Service, 2024), which makes most of them suitable for dairy cattle and for low production or maintenance level beef cattle (Suyama et al., 2007b), reflecting their high nutritional content.

### 4.2 Evaluation of lipids and their impact on forage quality, animal and human health

The TL content of the studied plants was in line with the generally low lipid levels of forages stated in the literature (< 8% DM; Davis et al., 2022). In recent years, there has been growing interest in under-exploited but promising plant species as alternative sources of vegetable oils. Some of these species can contain significant amounts of oil and, in addition, a high proportion of nutritionally desirable fatty acids, such as omega-3 PUFA (Manso et al., 2009; Timilsena et al., 2017). The PUFA levels recorded in our work were slightly higher than those determined by Cerci et al. (2011) in fresh alfalfa forages (30-50%), except in the case of *P. laevigata*, *E. decaisnei*, and *C. sventenii* (**Figure 4**). Previous studies have provided evidence of the differences in the FA profiles among various legumes, grasses, and herbaceous forage species, in which grasses tend to have higher levels of the C18 omega-3 PUFA, ALA than legumes, and conversely, lower contents of the C18 omega-6 PUFA, LA (Clapham et al., 2005; Elgersma et al., 2013; Whetsell and Rayburn, 2022). In this context, both C18 PUFA have been described to exert several benefits to animal health and well-being, and must be incorporated through the diet as they cannot be synthesized *de novo* (Morales-Almaráz et al., 2011; Davis et al., 2022). However, the increasing concern on the excessive intake of omega-6 fatty acids in the occidental diet, have raised the interest to search for plants and forage particularly rich in omega-3 fatty acids, with higher n-3/n-6 ratios, both for human and farmed animals feeding (Almeida et al., 2019; Zárate et al., 2017). It should also be stressed that *E. decaisnei* was the only species containing substantial amounts of two fatty acids of increasing nutraceutical value: GLA (0.8% of total FA) and SDA (2.3 of total FA). In this regard, species of the Echium genus are known to be rich sources of GLA and SDA (Guil-Guerrero et al., 2000a, b), two physiologically valuable FA for mammals’ health (López-Martínez et al., 2004; Wang et al., 2022; Berti et al., 2007), that have shown promising results in the cultivation of aquatic species (Díaz-López et al., 2009, 2010) and terrestrial farmed animals (Navarro et al., 2008).

It is well established that in the feeding of ruminants, the most important lipids are those containing fatty acids bound to glycerol (i.e., phospholipids, glycolipids and triglycerides). Glycerolipids are the most abundant type of lipid in our forages species, represented by major phospholipids including PC, phosphatidylserine (PS), phosphatidylinositol (PI), PG, and PE, and galactolipids such as SQDG, MGDG, and DGDG (Liu et al., 2017). Among the NLs, DAG and TAG were prominent. Like FAs, the relative proportions of these compounds fluctuate among plant species and are strongly influenced by the nutrient levels in the plants (Liu et al., 2017). SQDG, MGDG, and DGDG have been described as compounds with anti-inflammatory and antithrombotic properties (Ibañez and Cifuentes, 2013). Similarly, the levels of PTS in the forage can be very important as it has been demonstrated to reduce cholesterol levels in the form of low-density lipoprotein (LDL) in humans, thereby reducing the risk of cardiovascular diseases and inflammatory processes (Ibañez and Cifuentes, 2013; Kendel et al., 2015; Simonetti et al., 2021).

### 4.3 Implications of mineral composition in ruminant diets

The mineral composition of forage plant tissue shows significant variations, which could have direct implications on dietary formulations for ruminants. The levels of macro- and micro-minerals generally fall within the range that meets ruminants’ nutritional requirements. Species such as *C. viminalis*, *L. lancerottensis*, *E. decaisnei*, *L. acerifolia*, and *P. laevigata* had mineral contents that exceed the normally adequate values, particularly for Ca, Na, Fe and Cu (NRC, 2005). High Ca levels may cause health risks such as milk fever in dairy cows (Lean et al., 2006). The MTLs for dietary Ca in ruminants is 1.5% DM (NRC, 2005), yet *E. decaisnei* and *P. laevigata* recorded Ca concentrations of 22.7 and 34.2 g/kg DM, respectively, which can depress feed intake and milk yield. Sodium levels in some specimens reached 28 g/kg DM, potentially reducing appetite and increasing urine output, as well as the risk of kidney damage (ARC, 1980; NRC, 2007). Excessive dietary intake of Na is usually manageable as long as non-saline water is readily available for animals (Meyer et al., 1955; Nelson et al., 1955). Iron toxicity could lead to diarrhea and metabolic acidosis, in our case only *L. lancerottensis* exceeded the MTLs for this element (~ 500 mg/kg DM; NRC, 2005). Additionally, *E. decaisnei* exhibited Cu levels slightly above the safe threshold at 40 mg/kg DM (NRC, 2005), which could result in liver necrosis. Chronic Cu toxicity, particularly challenging to diagnose in cattle, involves symptoms such as hemoglobinuria and jaundice, typically more obvious in sheep, and is often indicated by high liver, kidney, and serum Cu concentrations (López-Alonso and Miranda, 2020).

Our results reveal strong interspecific variations in all parameters, indicating the potential benefits of using a diverse mix of species to optimize livestock nutritional intake. This is especially crucial in arid regions where plant species naturally exhibit unique nutritional profiles (Lee, 2018). Selecting appropriate forage species can optimize mineral intake and prevent deficiencies or toxicities that could impair livestock health and productivity.

Globally, most of the evaluated species have an adequate nutritional value, in addition to presenting a FA profile and certain lipid classes, such as PTS, beneficial for animal health and well-being. In particular, the species *B. bituminosa*, *P. coerulescens*, *E. decaisnei*, and *C. sventenii* could be promising candidates to be introduced as part of the diet of ruminants. The present work is limited to evaluating plant specimens under controlled conditions, so future research should focus on studying these species in field conditions, under different agronomic managements, that allow evaluating the influence of seasonal and environmental factors on the nutritional composition.

## 5 Conclusions

This study demonstrates that native and endemic plant species from arid environments like Fuerteventura Island have nutritional profiles capable of meeting the dietary requirements of livestock, making them viable forage options. The bromatological characterization of the twelve selected species shows they offer a sustainable alternative to conventional forages with potential nutritional benefits for livestock health and productivity. However, the strong interspecific variability suggests the combined use of different species as livestock feed.

By selecting native species with the most optimal nutritional profiles, it is possible to cultivate forages that not only withstand extreme conditions but also enhance livestock productivity and sustainability. This approach could be especially beneficial to regions heavily reliant on imported forages, thus reducing environmental impact and improving food security. Overall, this study provides a crucial foundation for the strategic selection and cultivation of native forage species in arid regions. Our findings advocate for an integrated approach that includes these native species in the agricultural matrix of arid regions, which could serve a dual purpose: enhancing livestock nutrition and conserving the unique botanical heritage of these ecosystems. Special attention should be paid to endemic species, some of which are endangered, as the discovery of potential benefits of their consumption in livestock health could contribute to their cultivation and simultaneously to their conservation. This paradigm shift toward the use of local and biodiverse plant species as forage represents a crucial step towards ecological sustainability, highlighting the untapped potential of arid region flora and paving the way for future agricultural innovations that address the nutritional needs of livestock in challenging environments.

## Data Availability

The original contributions presented in the study are included in the article/**Supplementary Material**. Further inquiries can be directed to the corresponding authors.
