# Supplementary material for: Extending beyond traditional forage: potential nutritional benefits of native plants in extreme arid insular regions
Source: Front Plant Sci. 2024 Dec 24;15:1476809. doi: 10.3389/fpls.2024.1476809 (PMC11703951; doi:10.3389/fpls.2024.1476809)
Supplement: Supplementary file 1 [file DataSheet1.docx]

Supplementary Material

**Figure S1.** Nursery of the “Pozo Negro” experimental farm dedicated to the reproduction of Fuerteventura native species with forage interest, cultivated from seed material collected from different locations on the island: germination trays with specimens of *Echium decaisnei* and *Bituminaria bituminosa* (A); specimens of *Asteriscus sericeus*, *Echium decaisnei*, *Bituminaria bituminosa*, and *Asparagus pastorianus* (B); specimens of *Echium decaisnei* (C) and *Lavandula canariensis* (D).

**Figure S2.** Thin layer chromatography of lipid classes from *Campylanthus salsoloides* (*Cs*) and *Bituminaria bituminosa* (*Bb*); cod roe standard (CR ST); a mixture of MGDG, DGDG and SQDG standards (Mix ST) and control (C) before (A) and after (B) thermal activation at 160°C. SE, sterol esters; TAG, triacylglycerols; UkNL, unknown neutral lipids; FFA, free fatty acids; PTS, phytosterols; DAG, diacylglycerols; P, pigments; MGDG, monogalactosyldiacylglycerol; UkPL, unknown polar lipids; DGDG, digalactosyldiacylglycerol; PE, phosphatidylethanolamine; SQDG, sulfoquinovosyl-diacylglycerol; PG, phosphatidylglycerol; PI, phosphatidylinositol; PS, phosphatidylserine; PC, phosphatidylcholine**.**

**Figure S3.** Fatty Acid Gas Chromatography of *Lotus lancerottensis* with internal standard (19:0).

**Table S1.** Main lipid class composition of the plant species grouped into three categories by family: Fabaceae, Poaceae, and a mixed group (Plantaginaceae, Brassicaceae, Boraginaceae, Malvaceae and Apocynaceae). Data are mean ± standard deviation (n=5); different letters in the same row indicate significant differences among species (p < 0.05).

**Table S2.** Main fatty acid composition of the plant species grouped into three categories by family: Fabaceae, Poaceae, and a mixed group (Plantaginaceae, Brassicaceae, Boraginaceae, Malvaceae and Apocynaceae). Data are mean ± standard deviation (n=5); different letters in the same row indicate significant differences among species (*p* < 0.05).

**Table S3.** Nutritional quality of FA of the plant species grouped into three categories by family: Fabaceae, Poaceae, and a mixed group (Plantaginaceae, Brassicaceae, Boraginaceae, Malvaceae and Apocynaceae). Data are mean ± standard deviation (n=5); different letters in the same row indicate significant differences among species (p < 0.05).

**Figure S1**

**
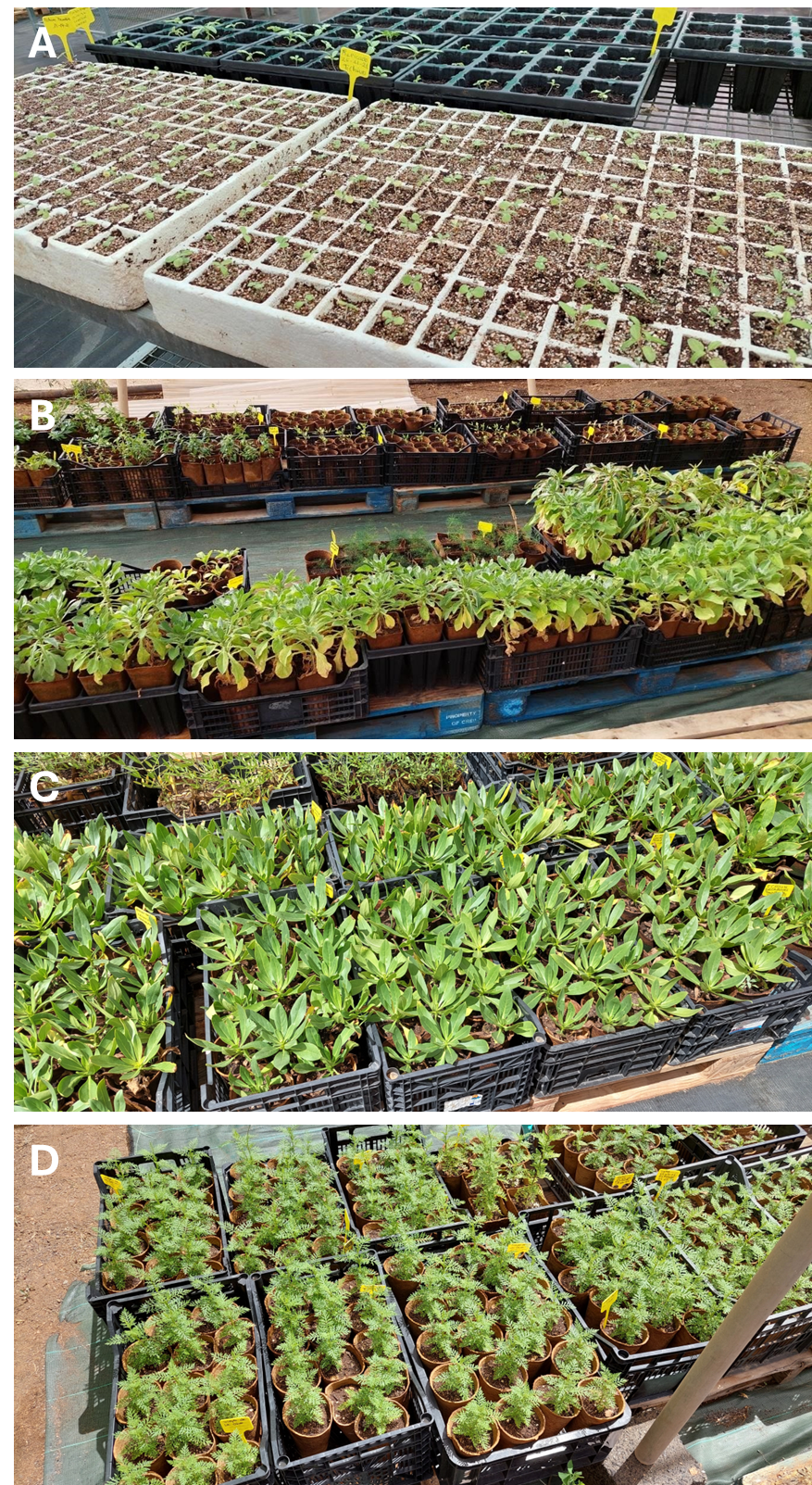
**

**Figure S2**

**
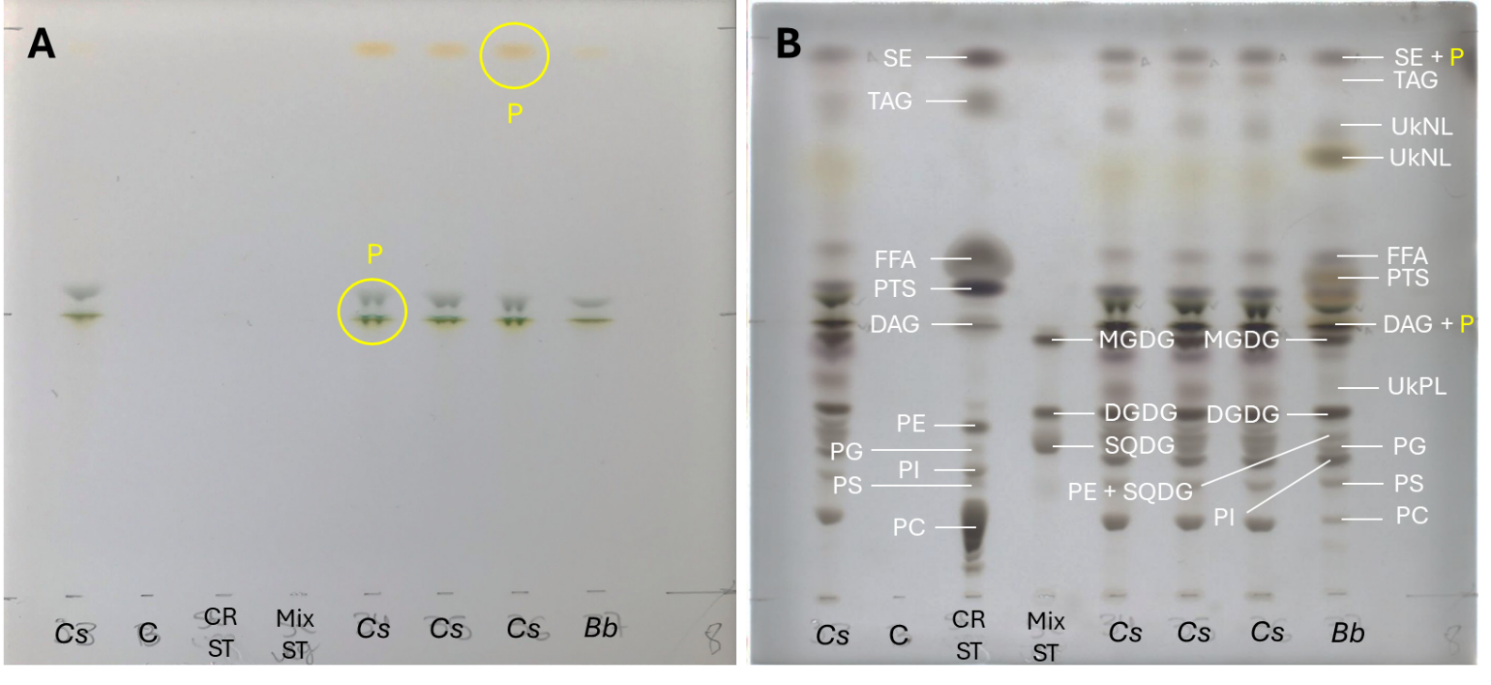
**

**Figure S3**

**
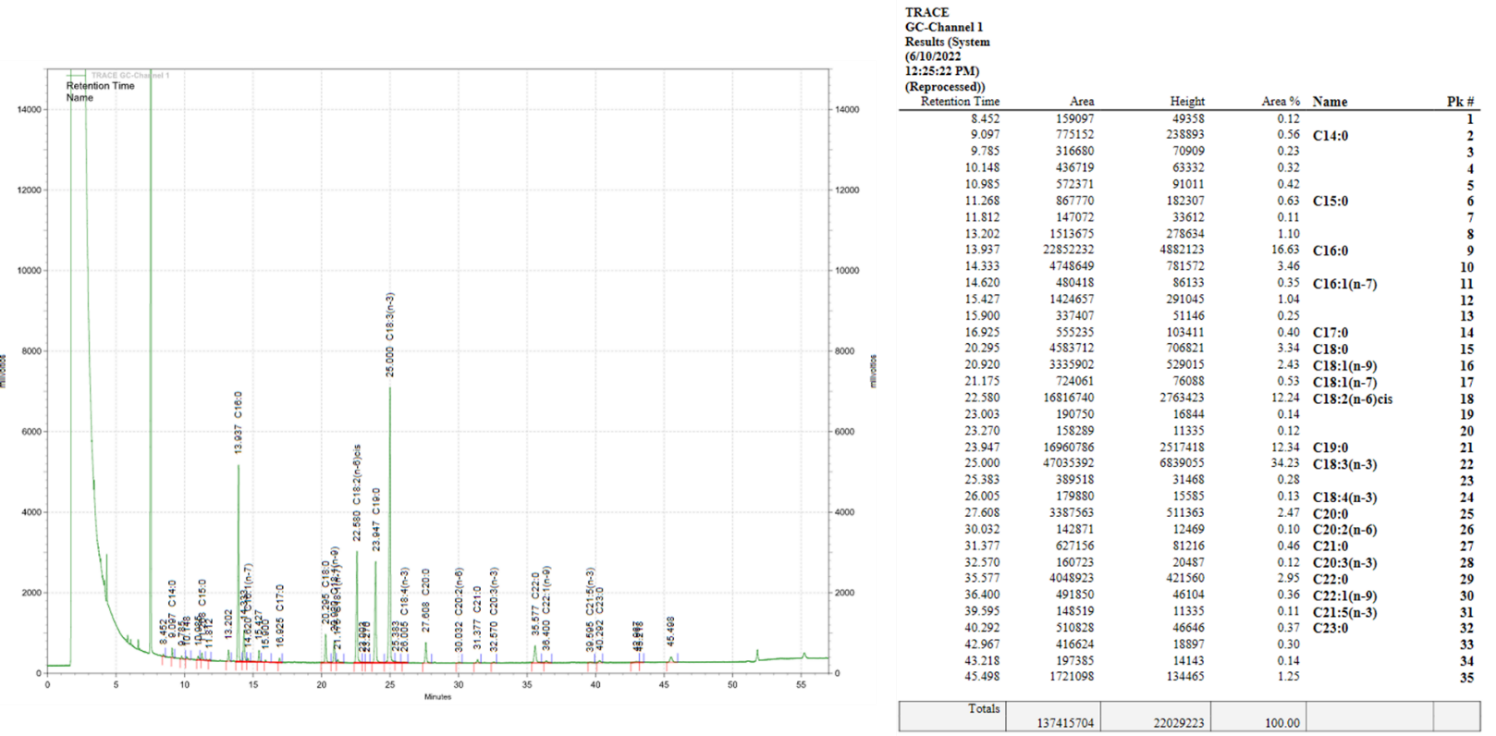
**

**Table S1**

| Lipid class  (% of TL) | *L. lancerottensis* | *B.*  *bituminosa* | *C.*  *viminalis* | *R.*  *rhodorhizoides* | *C.*  *ciliaris* | *P.*  *coerulescens* | *T.*  *teneriffae* | *L.*  *acerifolia* | *P.*  *laevigata* | *C.*  *salsoloides* | *E.*  *decaisnei* | *C.*  *sventenii* |
| --- | --- | --- | --- | --- | --- | --- | --- | --- | --- | --- | --- | --- |
| PC | 2.7 ± 1.2 | 2.3 ± 0.6 | 2.4 ± 0.4 | 2.7 ± 0.6 | 2.4 ± 0.3 a | 3.5 ± 0.7 ab | 4.0 ± 1.3 b | 1.5 ± 0.2 a | 2.5 ± 0.5 b | 5.6 ± 0.5 c | 3.9 ± 0.6 c | 1.9 ± 0.5 ab |
| PS + PI | 4.1 ± 0.5 b | 2.4 ± 0.4 a | 2.4 ± 0.4 a | 3.7 ± 0.9 b | 1.8 ± 0.1 a | 1.6 ± 0.2 a | 3.6 ± 0.5 b | 2.7 ± 0.3 b | 2.0 ± 0.5 a | 2.7 ± 0.2 ab | 4.9 ± 1.3 c | 2.4 ± 0.4 ab |
| PG | 3.2 ± 0.9 | 3.3 ± 0.3 | 4.2 ± 2.1 | 2.6 ± 0.1 | 3.3 ± 1.1 a | 8.6 ± 0.8 b | 4.6 ± 1.8 a | 2.3 ± 0.8 a | 4.1 ± 1.0 ab | 9.2 ± 0.5 b | 4.5 ± 1.3 c | 3.2 ± 0.1 ab |
| SQDG + PE | 12.0 ± 1.0 b | 9.0 ± 1.8 a | 11.2 ± 2.5 ab | 12.3 ± 0.8 b | 8.6 ± 4.0 | 7.5 ± 2.0 | 11.5 ± 2.3 | 7.7 ± 2.0 ab | 7.0 ± 0.9 ab | 5.3 ± 0.9 a | 8.5 ± 3.5 ab | 8.6 ± 1.3 b |
| DGDG | 13.0 ± 1.9 a | 10.4 ± 0.6 a | 13.3 ± 1.6 a | 17.9 ± 3.8 b | 10.9 ± 5.0 | 12.7 ± 1.0 | 12.4 ± 0.3 | 10.0 ± 2.7 | 9.3 ± 1.1 | 10.6 ± 0.7 | 8.8 ± 2.5 | 9.9 ± 1.0 |
| MGDG | 19.4 ± 3.8 b | 14.9 ± 0.9 a | 16.7 ± 1.4 ab | 15.6 ± 1.6 ab | 20.3 ± 5.2 b | 16.5 ± 1.4 b | 8.4 ± 0.8 a | 22.5 ± 3.4 b | 11.8 ± 2.6 a | 12.5 ± 1.0 a | 15.3 ± 3.0 a | 14.3 ± 0.6 ab |
| UkPL | 1.8 ± 0.6 | nd | 2.2 ± 0.2 | 1.7 ± 0.5 | nd | 8.1 ± 2.1 | 5.1 ± 0.5 | nd | 1.3 ± 0.4 | 12.9 ± 2.3 | 3.1 ± 0.7 | 1.8 ± 0.6 |
| TPL | 56.2 ± 1.3 b | 42.2 ± 0.5 a | 52.3 ± 0.9 b | 56.5 ± 1.2 b | 47.4 ± 2.2 a | 58.5 ± 0.7 b | 49.7 ± 1.3 a | 46.8 ± 1.3 bc | 38.0 ± 0.7 a | 58.7 ± 0.7 d | 49.0 ± 1.2 c | 42.1 ± 0.4 ab |
| DAG + P | 16.6 ± 4.1 ab | 21.4 ± 2.7 b | 20.6 ± 3.2 b | 12.6 ± 1.4 a | 13.8 ± 4.0 a | 20.9 ± 3.1 b | 18.3 ± 3.1 ab | 14.7 ± 3.2 a | 23.2 ± 2.3 b | 22.9 ± 1.4 b | 21.5 ± 5.2 b | 14.4 ± 3.7 a |
| PTS | 9.1 ± 1.1 b | 9.6 ± 1.0 b | 6.4 ± 0.7 a | 10.5 ± 2.0 b | 12.9 ± 2.8 b | 6.5 ± 0.9 a | 12.7 ± 3.4 b | 8.8 ± 2.5 ab | 5.8 ± 0.5 b | 7.0 ± 1.2 a | 8.8 ± 2.2 a | 16.7 ± 3.5 b |
| FFA | 5.9 ± 0.9 b | 7.3 ± 1.8 b | 2.1 ± 0.6 a | 6.5 ± 0.8 b | 7.1 ± 1.3 b | 3.5 ± 0.9 a | 4.5 ± 0.3 a | 6.1 ± 2.8 ab | 7.4 ± 1.0 b | 3.4 ± 0.5 a | 4.3 ± 1.9 a | 8.0 ± 0.8 b |
| TAG | 4.7 ± 0.7 | 3.7 ± 1.0 | 4.6 ± 0.8 | 4.3 ± 0.3 | 4.8 ± 0.9 ab | 2.2 ± 0.7 a | 5.8 ± 1.1 b | 8.6 ± 2.1 c | 1.4 ± 0.7 a | 2.1 ± 0.5 a | 4.4 ± 1.9 b | 4.6 ± 1.5 b |
| SE + P | 4.9 ± 0.9 a | 4.8 ± 0.5 a | 9.6 ± 1.6 b | 7.2 ± 0.3 c | 8.5 ± 2.5 | 7.7 ± 1.2 | 6.6 ± 2.1 | 7.9 ± 2.4 b | 9.6 ± 1.7 c | 4.6 ± 1.7 a | 6.8 ± 3.0 b | 8.4 ± 2.8 b |
| UkNL | 2.6 ± 0.7 | 11.0 ± 1.8 | 4.5 ± 1.2 | 2.5 ± 0.6 | 5.5 ± 2.7 | 0.7 ± 0.2 | 2.4 ± 0.4 | 7.1 ± 2.1 | 14.5 ± 1.8 | 1.4 ± 0.6 | 5.2 ± 1.3 | 5.7 ± 1.8 |
| TNL | 43.8 ± 1.3 a | 57.8 ± 0.8 b | 47.7 ± 1.0 a | 43.5 ± 0.7 a | 52.6 ± 0.9 b | 41.5 ± 1.0 a | 50.3 ± 1.3 b | 53.2 ± 0.4 bc | 62.0 ± 0.7 d | 41.3 ± 0.5 a | 51.0 ± 1.4 b | 57.9 ± 1.2 cd |

PC, phosphatidylcholine; PS, phosphatidylserine; PI, phosphatidylinositol; PG, phosphatidylglycerol; SQDG, sulfoquinovosyldiacylglycerol; PE, phosphatidylethanolamine; DGDG, digalactosyldiacylglycerol; MGDG, monogalactosyldiacylglycerol; UkPL, unknown polar lipids; TPL, total polar lipids; DAG, diacylglycerols; P, pigments; PTS, phytosterols; FFA, free fatty acids; TAG, triacylglycerols; SE, sterol esters; UkNL, unkown neutral lipids; TNL, total neutral lipids.

**Table S2**

| **Fatty acid  (% of total FA)** | ***L.  lancerottensis*** | ***B. bituminosa*** | ***C.  viminalis*** | ***R.  rhodorhizoides*** |
| --- | --- | --- | --- | --- |
| 14:0 | 0.7 ± 0.1 ab | 0.5 ± 0.3 a | 1.1 ± 0.2 b | 0.4 ± 0.1 a |
| 15:0 | 0.6 ± 0.1 b | 0.2 ± 0.1 a | 0.3 ± 0.0 a | 0.3 ± 0.0 a |
| 16:0 | 18.8 ± 2.0 b | 13.6 ± 0.4 a | 17.0 ± 1.1 b | 14.3 ± 0.5 a |
| 17:0 | 0.4 ± 0.1 b | 0.2 ± 0.0 a | 0.3 ± 0.1 a | 0.4 ± 0.0b |
| 18:0 | 3.6 ± 0.7 a | 3.1 ± 0.4 a | 5.9 ± 1.0 b | 3.5 ± 0.2 a |
| 20:0 | 3.1 ± 0.1 b | 1.3 ± 0.3 a | 1.1 ± 0.2 a | 4.3 ± 0.6 c |
| 21:0 | 0.4 ± 0.1 b | 0.2 ± 0.0 a | 0.1 ± 0.0 a | 0.2 ± 0.0 b |
| 22:0 | 3.4 ± 0.5 b | 2.5 ± 0.8 b | 1.2 ± 0.2 a | 2.8 ± 0.3 b |
| 23:0 | 0.5 ± 0.2 b | 0.3 ± 0.0 a | 0.2 ± 0.0 a | 0.5 ± 0.0 b |
| 24:0 | 1.6 ± 0.3 b | 2.7 ± 0.6 b | 0.5 ± 0.2 a | 2.0 ± 0.1 b |
| Ʃ SFA | 33.3 ± 3.6 b | 24.5 ± 1.6 a | 27.8 ± 2.4 a | 28.7 ± 0.5 a |
| 16:1 n-9 | 3.8 ± 0.6 a | 3.7 ± 0.4 a | 4.7 ± 0.4 b | 4.8 ± 0.2 b |
| 16:1 n-7 | 0.3 ± 0.1 ab | 0.2 ± 0.1 a | 0.5 ± 0.2b | 0.3 ± 0.0 a |
| 16:1 n-5 | 1.1 ± 0.2 a | 1.9 ± 0.2 c | 1.5 ± 0.1 b | 1.0 ± 0.0 a |
| 18:1 n-9 | 2.7 ± 0.3 | 2.3 ± 0.4 | 2.7 ± 1.7 | 2.3 ± 0.1 |
| 18:1 n-7 | 0.5 ± 0.1 | 0.4 ± 0.0 | 0.6 ± 0.1 | 0.4 ± 0.0 |
| 18:1 n-5 | nd | nd | 0.1 ± 0.0 | nd |
| 20:1 n-9 | 0.1 ± 0.0 | nd | nd | 0.1 ± 0.0 |
| 22:1 n-9 | 0.5 ± 0.3 b | 0.3 ± 0.1 ab | 0.1 ± 0.0 a | 0.1 ± 0.0 a |
| Ʃ MUFA | 9.1 ± 1.1 | 8.9 ± 0.7 | 10.2 ± 1.6 | 9.0 ± 0.3 |
| 16:3 n-4 | 0.1 ± 0.0 | nd | 0.1 ± 0.0 | 0.1 ± 0.0 |
| 18:2 n-6 (LA) | 15.1 ± 1.4 b | 9.6 ± 0.5 a | 10.9 ± 0.7 a | 21.6 ± 0.6 c |
| Ʃ n-6 PUFA | 15.1 ± 1.4 b | 9.6 ± 0.5 a | 10.9 ± 0.7 a | 21.6 ± 0.6 c |
| 18:3 n-3 (ALA) | 38.5 ± 4.0 a | 51.4 ± 3.3 b | 46.9 ± 3.5 b | 36.6 ± 0.9 a |
| 18:4 n-3 | 0.1 ± 0.0 | 0.2 ± 0.1 | 0.2 ± 0.1 | nd |
| Ʃ n-3 PUFA | 38.6 ± 4.1 a | 51.6 ± 3.2 b | 47.1 ± 3.6 ab | 36.6 ± 2.1 a |
| Ʃ PUFA | 53.8 ± 4.8 a | 61.3 ± 3.6 b | 58.1 ± 3.7 ab | 58.3 ± 0.6 ab |
| n-3/n-6 | 2.6 ± 0.3 b | 5.4 ± 0.3 d | 4.3 ± 0.4 c | 1.7 ± 0.1 a |
| UkFA | 3.8 ± 0.6 | 5.4 ± 1.7 | 3.9 ± 0.6 | 4.0 ± 0.4 |

(Table S2 continued)

| **Fatty acid  (% of total FA)** | ***C. ciliaris*** | ***P. coerulescens*** | ***T. teneriffae*** |
| --- | --- | --- | --- |
| 14:0 | 1.0 ± 0.3 b | 0.4 ± 0.0 a | 1.4 ± 0.6b |
| 15:0 | 0.7 ± 0.2 a | 0.4 ± 0.2 b | 0.3 ± 0.1 b |
| 16:0 | 17.0 ± 1.4 b | 13.1 ± 1.1 a | 19.6 ± 0.6 c |
| 17:0 | 0.4 ± 0.0 b | 0.1 ± 0.0 a | 0.6 ± 0.0 c |
| 18:0 | 2.9 ± 0.3 | 4.0 ± 1.9 | 4.0 ± 1.0 |
| 20:0 | 1.5 ± 0.4 b | 0.7 ± 0.2 a | 3.2 ± 0.2 c |
| 21:0 | 0.2 ± 0.0 | 0.1 ± 0.0 | 0.2 ± 0.0 |
| 22:0 | 2.6 ± 0.5 b | 0.7 ± 0.1 a | 2.0 ± 1.1 ab |
| 23:0 | 0.4 ± 0.1 b | 0.2 ± 0.0 a | 0.4 ± 0.0 b |
| 24:0 | 2.6 ± 0.4 c | 0.5 ± 0.1 a | 1.9 ± 0.1 b |
| Ʃ SFA | 29.3 ± 2.0 b | 20.3 ± 3.2 a | 33.7 ± 1.7 b |
| 16:1 n-9 | 4.4 ± 0.6 b | 6.0 ± 0.3 c | 2.5 ± 0.3 a |
| 16:1 n-7 | 0.2 ± 0.0 | 0.2 ± 0.0 | 0.3 ± 0.0 |
| 16:1 n-5 | 1.4 ± 0.3 b | 2.4 ± 0.1 c | 0.9 ± 0.1 a |
| 18:1 n-9 | 3.2 ± 1.0 | 4.0 ± 0.3 | 4.1 ± 0.7 |
| 18:1 n-7 | 0.4 ± 0.1 a | 0.3 ± 0.1 a | 0.8 ± 0.1 b |
| 20:1 n-11 | 0.1 ± 0.0 | nd | nd |
| 20:1 n-9 | 0.1 ± 0.0 a | 0.1 ± 0.0 a | 0.4 ± 0.0 b |
| 22:1 n-9 | 0.4 ± 0.0 b | 0.2 ± 0.0 a | 0.2 ± 0.0 a |
| Ʃ MUFA | 10.2 ± 0.9 a | 13.2 ± 0.7 b | 9.3 ± 1.1 a |
| 18:2 n-6 (LA) | 13.2 ± 2.3 b | 6.8 ± 0.2 a | 25.0 ± 2.2 c |
| Ʃ n-6 PUFA | 13.2 ± 2.3 b | 6.8 ± 0.2 a | 25.0 ± 2.2 c |
| 18:3 n-3 (ALA) | 41.8 ± 4.4 b | 54.5 ± 4.2 c | 27.1 ± 2.0 a |
| 18:4 n-3 | 0.2 ± 0.0 b | 0.1 ± 0.0 a | 0.2 ± 0.0 b |
| Ʃ n-3 PUFA | 42.0 ± 4.4 b | 54.6 ± 4.0 c | 27.2 ± 2.1 a |
| Ʃ PUFA | 55.2 ± 3.1 a | 61.3 ± 4.2 b | 52.2 ± 2.2 a |
| n-3/n-6 | 3.3 ± 1.0 b | 8.1 ± 0.5 c | 1.1 ± 0.2 a |
| UkFA | 5.4 ± 0.6 | 5.2 ± 1.0 | 4.8 ± 1.5 |

| **Fatty acid  (% of total FA**) | ***L. acerifolia*** | ***P. laevigata*** | ***C.  salsoloides*** | ***E. decaisnei*** | ***C. sventenii*** |
| --- | --- | --- | --- | --- | --- |
| 14:0 | 2.1 ± 0.2 b | 1.2 ± 0.6 ab | 0.4 ± 0.1 a | 2.4 ± 3.8 ab | 0.9 ± 0.3 ab |
| 15:0 | 0.4 ± 0.2 | 0.5 ± 0.2 | 0.2 ± 0.0 | 0.6 ± 0.5 | 0.6 ± 0.1 |
| 16:0 | 17.6 ± 1.6 ab | 20.5 ± 1.7 b | 16.5 ± 1.1 a | 16.0 ± 2.8 a | 15.4 ± 0.5 a |
| 17:0 | 0.3 ± 0.0 a | 0.3 ± 0.0 b | 0.3 ± 0.0 ab | 0.4 ± 0.1 b | 0.4 ± 0.0 b |
| 18:0 | 2.0 ±0.2 a | 6.7 ± 1.3 c | 4.3 ± 1.3 a | 4.1 ± 0.7 ab | 5.8 ± 1.6 bc |
| 20:0 | 0.9 ± 0.1 b | 1.4 ± 0.1c | 0.5 ± 0.1 a | 5.4 ± 0.8 d | 1.1 ± 0.1 bc |
| 21:0 | 0.2 ± 0.0 ab | 0.2 ± 0.0 ab | 0.1 ± 0.0 a | 0.2 ± 0.0 b | nd |
| 22:0 | 0.7 ± 0.1 a | 0.9 ± 0.1 a | 0.7 ± 0.1 a | 8.9 ± 1.8 b | 0.5 ± 0.0 a |
| 23:0 | 0.2 ± 0.0 ab | 0.2 ± 0.0 a | 0.3 ± 0.0 bc | 0.4 ± 0.1 c | nd |
| 24:0 | 0.8 ± 0.1 a | 0.7 ± 0.1 a | 0.8 ± 0.1 a | 5.1 ± 1.2 b | 0.6 ± 0.1 a |
| Ʃ SFA | 25.3 ± 2.0 a | 32.6 ± 3.5 b | 24.3 ± 2.7 a | 43.6 ± 6.5 c | 25.3 ± 1.9 a |
| 16:1 n-9 | 3.9 ± 0.5 a | 3.9 ± 0.6 a | 5.3 ± 0.6 b | 4.0 ± 0.9 ab | 5.1 ± 0.7 b |
| 16:1 n-7 | 0.3 ± 0.0 | 0.5 ± 0.1 | 0.3 ± 0.0 | 1.3 ± 0.3 | 0.3 ± 0.0 |
| 16:1 n-5 | 1.0 ± 0.0 b | 1.8 ± 0.3 d | 0.4 ± 0.1 a | 1.4 ± 0.1 cd | 1.1 ± 0.1 ab |
| 17:1 n-7 | nd | nd | nd | nd | 6.8 ± 0.6 |
| 18:1 n-9 | 1.3 ± 0.2 a | 7.1 ± 0.8 c | 5.9 ± 0.6 bc | 7.4 ± 3.0 c | 4.4 ± 0.4 b |
| 18:1 n-7 | 0.5 ± 0.1 ab | 0.7 ± 0.1 b | 0.5 ± 0.1 ab | 0.7 ± 0.2 b | 0.4 ± 0.0 a |
| 20:1 n-9 | nd | nd | 0.1 ± 0.0 a | 0.2 ± 0.0 b | nd |
| 22:1 n-9 | 0.2 ± 0.0 ab | 0.2 ± 0.0 abc | 0.1 ± 0.0 a | 0.4 ± 0.1 c | 0.3 ± 0.0 bc |
| Ʃ MUFA | 7.1 ± 0.7 a | 14.1 ± 1.5 b | 12.6 ± 0.8 b | 15.3 ± 4.4 bc | 18.4 ± 1.0 c |
| 16:3 n-4 | 0.3 ± 0.0 c | 0.1 ± 0.0 ab | 0.1 ± 0.0 a | 0.2 ± 0.0 c | 0.1 ± 0.0 ab |
| 18:3 n-4 | nd | nd | 0.4 ± 0.0 | 0.2 ± 0.0 | nd |
| 18:2 n-6 (LA) | 16.8 ± 0.9 d | 13.6 ± 1.2 c | 9.5 ± 1.0 b | 6.4 ± 2.1 a | 17.9 ± 1.1 d |
| 18:3 n-6 (GLA) | nd | nd | nd | 0.8 ± 0.2 | nd |
| Ʃ n-6 PUFA | 16.8 ± 0.9 c | 13.6 ± 1.2 b | 9.5 ± 1.0 a | 7.2 ± 2.3 a | 17.9 ± 1.1 c |
| 18:3 n-3 (ALA) | 45.8 ± 3.5 c | 33.6 ± 3.7 b | 47.9 ± 3.8 c | 24.8 ± 7.8 a | 28.2 ± 2.3 ab |
| 18:4 n-3 | 0.2 ± 0.0 a | 0.2 ± 0.0 a | 0.2 ± 0.0 a | 2.3 ± 0.3 b | 0.3 ± 0.0 a |
| Ʃ n-3 PUFA | 46.0 ± 3.5 b | 33.8 ± 3.8 a | 48.2 ± 3.7 b | 27.1 ± 9.0 a | 28.5 ± 2.2 a |
| Ʃ PUFA | 63.1 ± 3.0 c | 47.6 ± 4.5 b | 58.1 ± 3.6 cd | 34.7 ± 10.0 a | 46.5 ± 2.7 bc |
| n-3/n-6 | 2.8 ± 0.3 ab | 2.5 ± 0.2 b | 5.1 ± 0.7 c | 3.9 ± 1.3 bc | 1.6 ± 0.2 a |
| UkFA | 4.5 ± 0.4 | 5.7 ± 1.1 | 5.0 ± 0.4 | 6.3 ± 1.0 | 9.8 ± 1.0 |

(Table S2 continued)

SFA, saturated fatty acids; MUFA, monounsaturated fatty acids; LA, linoleic acid; GLA, gamma-linolenic acid; PUFA, polyunsaturated fatty acids; ALA, alpha-linolenic acid; UkFA, unknown fatty acids; nd, not detected.

**Table S3**

| **Species** | **IA** | **IT** | **hH** |
| --- | --- | --- | --- |
| *B. bituminosa* | 0.27 ± 0.02 a | 0.10 ± 0.01 a | 0.85 ± 0.04 b |
| *C. viminalis* | 0.40 ± 0.06 b | 0.15 ± 0.02 b | 0.77 ± 0.12 b |
| *L. lancerottensis* | 0.40 ± 0.07 b | 0.18 ± 0.04 b | 0.92 ± 0.14 a |
| *R. rhodorhizoides* | 0.29 ± 0.01 a | 0.14 ± 0.01 a | 1.68 ± 0.07 c |
|  |  |  |  |
| *C. ciliaris* | 0.37 ± 0.05 b | 0.15 ± 0.03 a | 0.91 ± 0.12 a |
| *P. coerulescens* | 0.25 ± 0.05 a | 0.10 ± 0.02 a | 0.81 ± 0.08 a |
| *T. teneriffae* | 0.47 ± 0.06 c | 0.25 ± 0.02 b | 1.42 ± 0.11 c |
|  |  |  |  |
| *C. salsoloides* | 0.32 ± 0.05 a | 0.13 ± 0.02 a | 0.91 ± 0.09 b |
| *C. sventenii* | 0.37 ± 0.09 b | 0.20 ± 0.03 b | 1.44 ± 0.10 c |
| *E. decaisnei* | 0.62 ± 0.39 c | 0.25 ± 0.11 c | 0.79 ± 0.25 a |
| *L. acerifolia* | 0.40 ± 0.04 b | 0.14 ± 0.02 a | 0.93 ± 0.05 b |
| *P. laevigata* | 0.53 ± 0.10 c | 0.24 ± 0.05 b | 0.97 ± 0.11 c |

IA, index of atherogenicity; IT, index of thrombogenicity; hH, hypocholesterolemic/hypercholesterolemic fatty acids ratio.
